# Associations between Food Group Intake, Cognition, and Academic Achievement in Elementary Schoolchildren

**DOI:** 10.3390/nu11112722

**Published:** 2019-11-09

**Authors:** Rachel Bleiweiss-Sande, Kenneth Chui, Catherine Wright, Sarah Amin, Stephanie Anzman-Frasca, Jennifer M. Sacheck

**Affiliations:** 1 Johns Hopkins Bloomberg School of Public Health, Department of Health, Behavior & Society, 615 North Wolfe Street, Baltimore, MD 21218, USA; 2 Tufts University Department of Public Health and Community Medicine, Department of Public Health & Community Medicine, 136 Harrison Avenue, Boston, MA 02111, USA; kenneth.chui@tufts.edu; 3 Friedman School of Nutrition Science & Policy at Tufts University, 75 Kneeland Street, Boston, MA 02111, USA; catherine.wright@tufts.edu; 4 University of Rhode Island, Fogarty Hall, 41 Lower College Road, Kingston, RI 02881, USA; sarah_amin@uri.edu; 5 University at Buffalo Jacobs School of Medicine and Biomedical Sciences, Division of Behavioral Medicine, G56 Farber Hall, 3435 Main Street, Buffalo, NY 14214, USA; safrasca@buffalo.edu; 6 Milken Institute of Public Health, The George Washington University, 950 New Hampshire Ave, NW, Washington, DC 20052, USA; jsacheck25@gwu.edu

**Keywords:** children, diet, food groups, cognition, academic achievement, nutrition, low-income

## Abstract

Nutrition plays an important role in proper physical and cognitive functioning. However, there is limited evidence on the relationship between overall diet, cognition, and academic success in children, particularly among low-income and diverse groups. The objective of this study was to examine the relationships between healthful versus less healthful food group intake, cognitive performance, and academic achievement in a diverse sample of schoolchildren. 868 urban schoolchildren (age 8 to 10 years) participated in the study. Intake of healthful (fruits, vegetables, unsweetened beverages) and less healthful (sweet and salty snacks, sugar-sweetened beverages) food groups was determined via a food frequency questionnaire. Digit Span and Stroop test scores were used to assess cognitive performance. Academic achievement was assessed via standardized test scores. Multiple Poisson and multiple linear regression were used to test the associations between diet and cognitive scores. Multiple ordered logistic regression was used to assess the associations between diet and academic achievement. Potential confounders (age, sex, body mass index (BMI) z-score, race/ethnicity, English language learner status, individualized education plan enrollment, physical activity, and parent education level) were tested for inclusion in all models. The sample included 868 children (56.7% girls; 33.2% non-Hispanic white, 26.2% Hispanic, 17.1% multiracial/other, 8.3% non-Hispanic black; 40.5% overweight/obese). The most frequently consumed foods were fruits and sweet snacks (1.9 and 1.6 servings per day, respectively). There were no statistically significant associations between diet and cognitive test scores. Greater intake of less healthful food groups (sweet snacks, salty snacks, and sweetened beverages) was associated with lower math (OR = 0.91, CI [0.84, 0.98], *p* = 0.014) and English standardized test scores (OR = 0.87, CI [0.80, 0.94, *p* = 0.001). Greater intake of sweet snacks and fruits was associated with lower English scores (OR = 0.72, 95% CI [0.59, 0.88] *p* = 0.001; and OR = 0.75, 95% CI [0.72, 0.94] *p* = 0.003, respectively). Consumption of less healthful food groups was associated with poorer academic achievement. Further research may shed light on unexpected associations between fruit consumption and achievement. Policies targeting multiple dietary components may positively influence child academic achievement and development.

## 1. Introduction

Cognition is a set of higher mental functions including memory, learning, and attention; it is demonstrated to be a significant predictor of a child’s academic achievement and his/her future quality of life [1,2]. Nutrition plays an important role in proper physical and cognitive functioning, particularly during childhood. Recent evidence demonstrates that children in the United States (U.S.) are not meeting dietary recommendations [3,4,5]. It has been suggested that nutrient insufficiency in children may be due to dietary patterns rich in sweet and salty snack foods and sugar-sweetened beverages (SSBs) and low in fruits, vegetables, and whole grains [4,6]. It is widely accepted that individual foods and nutrients may have interactive and potentially cumulative effects on health. For this reason, the 2015–2012 Dietary Guidelines for Americans shifted the focus of its recommendations from foods and nutrients to overall eating patterns [3]. While there is a substantial body of evidence concerning the association between single micronutrients or foods and cognition, there is limited evidence on the relationship between overall diet and mental capacity in children [7,8,9,10]. In particular, more research is needed on the impact of food groups on cognition and academic success in children to inform effective intervention strategies [10,11].

Recent findings from neuroscientific research point to an interplay between diet and cognition independent of weight status and physical activity [12]. Several studies in animal models and adults demonstrate that “Western diets”—intake patterns abundant in refined carbohydrates including sugar and saturated fat—can have a negative effect on learning and memory [13,14,15], while intake of foods with high levels of flavonoids and antioxidants, such as fruits and vegetables, have been shown to ameliorate cognitive impairment [14]. In children, there is ample evidence regarding the role of single micronutrients or foods; however, results are frequently inconclusive or conflicting [16,17,18,19,20,21]. In addition, single nutrients may have synergistic or antagonistic effects in different contexts, suggesting that overall diet or consumption of specific food groups may be more effective targets for public health interventions [10,22]. 

There is more consistent, but limited evidence on the role of healthful versus less healthful food group intake and cognition in children [10]. One systematic review found overall weak, positive associations between healthful diets (patterns rich in fresh fruits, vegetables and whole grains) in early life and intelligence quotient (IQ) in later childhood and weak, negative associations between discretionary eating patterns (soda, sweets, and refined grains) and IQ [23]. Notably, the review’s study population did not include a wide range of racial and socio-economic backgrounds, limiting generalizability. Potential modifying factors in the relationship between diet and cognition have also emerged. There is some evidence that gender, age, parental education, household income [24,25], weight status, physical activity [9,23,26,27], and breakfast consumption [28] may play a role, but there is limited research examining these factors simultaneously. 

### Diet and Academic Achievement

Most studies examining the relationship between dietary intake and academic achievement have focused on specific foods or nutrients, with relatively few examining the relationships between consumption of foods groups and academic performance in children. Breakfast consumption has emerged as a positive predictor, and fast food and sweetened beverage consumption as negative predictors of academic achievement in a 2017 systematic review [29]. Notably, the majority of studies included did not use a validated dietary assessment method, and most considered a single aspect of diet, such as breakfast or individual nutrients instead of multiple dietary behaviors [29]. In addition, the 2017 review found that only 10 of the 40 studies examined included ethnicities other than Caucasian, and only 1 studied was done in an exclusively low-income population [29]. A later study found a positive relationship between vegetable consumption and verbal test scores, and a negative association between SSBs and math scores [30]. Similar studies have found weak, positive associations between diet quality scores and academic performance [26,31,32,33].

Even with the demonstrated link between diet, cognition, and academic achievement, evidence regarding the impact of consumption of food groups alone and in combination is still lacking, and there is a paucity of research examining diet and academic performance in lower-income and diverse schoolchildren. There is also a need for research that examines multiple factors related to diet and school performance concurrently, using rigorous, objective measures. Therefore, the aim of this study was to investigate associations between intake of healthful and less healthful food groups, cognitive measures, and academic achievement among a diverse, lower-income population of elementary schoolchildren participating in the Fueling Learning Through Exercise (FLEX) Study. 

## 2. Methods

This study is a sub-analysis of baseline data from the FLEX study, a cluster randomized controlled trial designed to evaluate the impact of two school-based physical activity programs on activity levels, as well as cognitive performance and academic achievement among lower-income and racially/ethnically diverse elementary schoolchildren in Massachusetts [34]. In brief, all 3rd and 4th graders (8 to 10 years old) from participating schools were eligible to enroll at baseline of the two-year study during the 2015–2016 school year [34]. FLEX study recruitment took place during two waves. Participants from wave one completed an alternate dietary screener and thus were not included in the present study [34]. A total of 1008 children from 8 school districts and 18 schools were enrolled during wave two recruitment [34]. Of these children, 21 moved out of the school district or were absent from school during baseline data collection, for a total of 987 included in the current analysis. Apart from one school from a district with 34% low-income students, all enrolled districts had greater than 40% student eligibility for free- or reduced-price lunch and/or 40% racial/ethnic minority students [34]. All participants were required to provide written assent and have a parent or caregiver provide written, informed consent. The Tufts Institutional Review Board (IRB) as well as individual school district IRBs approved the study. 

### 2.1. Demographics. 

The FLEX study data collection procedures are described in detail elsewhere [34]. Child’s date of birth, age at time of enrollment, sex, race/ethnicity and parent education level were collected through a self-reported questionnaire by a parent or guardian at baseline. Child’s height and weight were collected at baseline by trained research assistants according to standard protocol [34]. BMI Z-score was calculated as weight in kilograms per height in meters squared, adjusted for child age and sex per the Centers for Disease Control and Prevention BMI-for-age reference charts [35]. BMI percentiles were classified as underweight (<5th percentile), normal weight (5th–85th percentile), overweight (85th–95th percentile), and obese (≥95th percentile) according to accepted cut points for use in descriptive analyses [36]. 

### 2.2. Diet 

Dietary intake was assessed by child self-report using the validated FLEX Food Frequency Questionnaire (FLEX FFQ) [37], a paper and pencil questionnaire adapted from several validated food frequency questionnaires [38,39,40]. Questionnaires were administered in small groups with the help of a research assistant. The FLEX FFQ includes 39 questions about the frequency (number of times in the past week) and amount (a little, some, a lot) of foods commonly consumed by the study population. Portion sizes are matched to standard serving size equivalents (a little = ½ serving; some = 1 serving; a lot = 1.5 servings). The questionnaire is not designed to assess total dietary intake, instead asking about how often categories of foods that are more healthful (fruits, vegetables, milk, water) and those that should be limited (sweetened beverages, salty snacks, sweet snacks) are consumed [3,37]. Daily serving sizes are calculated by dividing the total reported days the item was consumed by the reported number of servings. A breakfast questionnaire was used to assess whether students consumed breakfast on the day of the cognitive tests and whether they typically eat breakfast on weekdays.

### 2.3. Cognitive Assessments

Cognitive assessments were administered by trained research assistants one-on-one in quiet areas during the school day. The Digit Span (DS) Forwards and DS Backwards are widely used measures of attention, concentration, executive functioning, and short-term memory in children [41,42]. The test involves listening to a sequence of numbers read aloud and repeating back the sequence verbatim (DS Forwards) or in reverse order (DS Backwards). When the sequence is repeated back correctly, a new sequence one digit longer is presented. After an incorrect response, the child is given a second attempt with a different sequence of the same length. Scores reflect the longest sequence correctly repeated back by the child. While both tasks are used to assess short-term memory, with higher scores indicating improved memory processes [41], the DS Forwards is most sensitive to measuring short-term memory and sequencing while the DS Backwards best captures deficits in working memory [41,43]. Therefore, the DS Forwards and DS Backwards scores are used as separate, discrete-count outcome variables in analyses.

The Stroop color-word task is frequently used to assess attention and impulse control in child and adolescent populations [44,45,46]. In the FLEX study, a congruent and incongruent task were administered, each lasting 45 seconds. In the congruent task, the participant is given a card with 100 color-words (red, orange, yellow, green, blue, black, purple) printed in the same color ink as the word. They are asked to read aloud, in order, as many words as they can in 45 seconds. The second task presents a set of 100 color-words printed in a different color ink than the word (such as the word “green” printed in red ink), and the child is asked to say aloud the color of the ink, identifying as many as possible in sequence in 45 seconds. To complete the incongruent task, participants must inhibit a prepotent response (i.e., reading the printed word) and instead initiate a normally inhibited response to say the ink color [7,44,46]. To assess inhibitory control independent of a participant’s reading or speaking ability, a ratio of Stroop incongruent score to Stroop congruent score was used as an outcome variable in analyses. Higher ratio scores indicate a better ability to override interference or the distraction caused by the contrasting word and color [45].

### 2.4. Academic Achievement 

Massachusetts Comprehensive Assessment System (MCAS) standardized test scores provided by the Massachusetts Department of Elementary and Secondary Education were used as an indicator of academic achievement. MCAS is a state-wide standards-based student assessment program for all elementary and high school students attending public school in the Commonwealth of Massachusetts [47]. Test results are used to evaluate student, school, and district performance according to the Massachusetts curriculum frameworks learning standards [47].

Achievement levels are reported on the MCAS according to four categories: advanced, proficient, needs improvement, and warning. The cut-off for each level is determined by the minimum score needed to attain an achievement level, which applies across subject areas and grade. MCAS scores and achievement level are reported separately for math and English language arts. Because the MCAS mathematics and English language arts exams were administered to 4th graders and older during the 2014–2015 school year and the FLEX study enrolled 3rd and 4th graders at baseline, scores from 4th graders only were available for this study.

Information on English language learner status (yes/no) was collected as part of the MCAS test. Therefore, this information was only available for the subset of 4th graders who were eligible to take the exam.

### 2.5. Physical Activity 

Waist-worn accelerometers (GT3X+, Actigraph LLC, Pensacola, FL, USA) were used to measure physical activity levels among participants in the FLEX study. Participants were instructed to wear accelerometers during all waking hours for seven consecutive days, except while swimming or bathing. Therefore, physical activity levels for those children who swam during the week may be underestimated. Mean daily minutes of physical activity levels (sedentary, light, moderate, vigorous) were assessed according to thresholds developed for children [48]. Children with at least 3 days and 10 hours of wear time per day were included in the analyses. A detailed physical activity assessment protocol is described elsewhere [34].

### 2.6. Statistical Analysis 

All statistical analyses were performed using Stata version 15 (StataCorp; College Station, TX, USA). Descriptive statistics were first calculated for the study sample. 

#### 2.6.1. Dietary Intake

Average servings per day of food groups as reported on the FLEX FFQ were used as continuous, independent variables in regression models testing the association between dietary intake, cognitive measures, and MCAS scores. Healthful foods were operationalized as total daily average servings of foods and beverages to encourage (fruits, vegetables, water, milk, and 100% fruit juice), while less healthful foods were operationalized as foods and beverages to limit (salty snacks such as chips, sweet snacks such as candy, soft drinks, sports drinks, and flavored juice drinks), as described in previous works [3,37]. Relationships between total reported intake in servings per day of individual food groups (fruit, vegetables, salty snacks and sweet snacks, unsweetened beverages, SSBs) and the outcomes of interest were also examined. 

#### 2.6.2. Dietary Intake and Cognitive Outcomes

The relationship between intake of the healthful and less healthful food groups overall, as well as individual food groups, and DS Forwards and DS Backwards was assessed using Poisson regression. Poisson regression was chosen to account for the fact that scores were discrete, but not categorical variables [49]. Unadjusted and adjusted multivariate linear regression models were fit to test the relationship between food groups and Stroop test score ratio. Potential confounders in the association between dietary pattern and cognitive measure were identified from relevant literature and were individually tested for predictive power using a log likelihood test [8,23,24,50,51,52]. These included age, sex, BMI for age Z-score, race/ethnicity, maternal and paternal education level, breakfast consumption on the day of the tests, and mean moderate-to-vigorous physical activity levels (MVPA). We used backwards elimination to exclude confounders and establish the final models [53]. Although the Stroop and digit span tests measure different types of cognitive function, they share the same broad categories of confounders [42].

#### 2.6.3. Dietary Intake and Academic Achievement 

Academic achievement was operationalized as four discrete categories (advanced, proficient, needs improvement, and warning). Because the mean point difference between categories may vary and the categories contain a hierarchy, ordered logistic regression was used to examine the relationship between diet, MCAS math, and English language arts scores [49]. We ran a test of proportional odds for each model to ensure that the assumptions of ordered logistic regression were upheld [49]. Potential confounders, including age, sex, BMI for age Z-score, race/ethnicity, maternal and paternal education level, and mean MVPA were identified from similar studies and were tested for predictive power [12,26,29,30,32,33]. Since breakfast questionnaires were not administered on the MCAS testing day, typical reported breakfast consumption (yes/no) was used as a covariate in academic achievement models. English language learner status, as reported on the MCAS, was also included as a potential covariate in academic achievement models; p-values less than 0.05 were considered statistically significant. 

## 3. Results

### 3.1. Final Sample

Participants who had missing data from any food or beverage category (*n* = 38), or the DS Forwards, DS Backwards, Stroop congruent or incongruent score (*n* = 10), were not included in any of the analyses. Daily reported servings of food groups greater than 4 standard deviations above the mean were considered outliers and were also not included in the analysis (*n* = 71), for a final sample size of 868. Independent Student’s t-tests were run to test whether subjects dropped because outliers were systematically different from other participants. We found no significant differences between the dropped sample and the main sample for any demographic variable, indicating that this group was a random sample of the total population.

Of the students with complete dietary data, 475 were 4th graders who were eligible to take the MCAS tests. Students missing either the mathematics or English language arts MCAS score from this subsample (*n* = 45) were dropped, for a final sample size of 430 students included in the diet and academic achievement analyses. We ran additional models to test associations with diet and cognitive outcomes in this subset of only 4th grade students in order to have matched samples for comparison with the academic achievement results.

### 3.2. Descriptive Statistics

Table 1 includes demographic information for both the entire sample of 3rd and 4th graders (*n* = 868; 57% female) and the sub-sample of 4th-grade children that completed the MCAS assessment (*n* = 430). The overall study population included a high percentage of overweight and obese students (40.6%), approximately one-quarter of which was Hispanic. Roughly one-third of mothers and 43% of fathers reported an education level corresponding to high school or less. Within the subset of students who took the MCAS assessment, 21% were English language learners.

### 3.3. Dietary Intake.

The highest reported mean servings of foods per day were fruits (1.9 servings), followed by sweet snacks (1.6 servings; Table 2). Soft drinks and fruit juice were the lowest reported mean servings consumed (0.3 and 0.4 servings, respectively). Most children (89%) reported eating breakfast on the day of cognitive testing and typically eating breakfast on weekdays (91%). 

### 3.4. Dietary Intake and Cognitive Outcomes

Mean DS Forwards and DS Backwards scores were 4.7 (SD = 1.09) and 2.8 (SD = 1.01), respectively. Age was positively and significantly associated with a higher DS score in all models (*p* < 0.01). Combined categories of healthful foods (fruits, vegetables, and unsweetened beverages) and less healthful foods (salty and sweet snacks and SSBs) were non-significant predictors of digit span score in unadjusted (not shown) and age-adjusted models (Table 3). All individual food groups were non-significant predictors of DS Forwards and DS Backwards scores.

The mean ratio of incongruent to congruent Stroop test score was 0.4 (SD = 0.1). Higher BMI Z-score was associated with lower Stroop test score, and greater MVPA was associated with higher Stroop test score in all models (*p* < 0.01). Combined food categories and individual foods groups were non-significant predictors of the Stroop score in unadjusted models (not shown) and in models adjusted for BMI Z-score and MVPA (Table 4).

### 3.5. Dietary Intake and Academic Achievement

Over 70% of students scored in the proficient or advanced categories on the mathematics assessment, while 58% scored in these two categories in English language arts (Table 1). Female sex, English language learner status, individualized education plan (IEP) enrollment and higher BMI-z score were associated with lower math score, and higher paternal education was associated with higher math score in all models (*p* < 0.01). English language learner status and IEP enrollment were associated with lower English score, and higher maternal education was associated with higher English score in all models (*p* < 0.01). Less healthful food intake was associated with lower math (OR = 0.91, 95% CI = 0.84, 0.98) and English (OR = 0.87, 95% CI = 0.80, 0.94) scores, with the greatest effect seen for English score (Table 5). Higher reported intakes of fruits (OR = 0.75, 95% CI = 0.62, 0.91) and sweet snacks (OR = 0.72, 95% CI = 0.59, 0.88) were associated with lower English score after adjustment for sex, English language learner status, enrollment in IEP, mother’s education level, and BMI Z-score. For a one-serving increase in sweet snacks, the odds of scoring in the advanced category compared to the lower categories on the English assessment decreased by 28 percent. There were no significant associations between individual food groups and math score. 

## 4. Discussion

There is a major gap in the literature regarding the relationship between dietary intake and cognition/academic achievement in socioeconomically disadvantaged children. This study examined the relationships between intake of more and less healthful foods commonly consumed by children from diverse, lower-income school districts in MA and cognitive/academic outcomes, taking into consideration multiple related factors including BMI, physical activity levels, breakfast consumption, behavioral issues, and socioeconomic variables. Our findings demonstrate that greater overall intakes of less healthful foods (sweet and salty snacks and SSBs) are associated with lower math and English standardized test scores, suggesting that dietary patterns rich in energy-dense, nutrient-poor foods are linked to lower academic achievement in this population. 

These results are consistent with those reported in a systematic review by Burrows et al., which demonstrated a negative association between “junk foods” or SSBs and academic achievement in school children [29]. Notably, the majority of participants examined were from higher-income households, and socioeconomic factors were controlled for in only a small group of the studies. Our findings support the consideration of several covariates when examining diverse groups of children, including English language learner status, IEP enrollment, and parental education. In contrast to the results of Burrows et al., we did not find an association between achievement and breakfast consumption [29]. Since most (~91%) of FLEX 4th-grade participants reported typically eating breakfast, it is likely that our study was underpowered to detect an effect related to breakfast consumption. 

Contrary to studies that demonstrate a weak but positive association between dietary intake of food groups and cognitive performance, we found no significant relationships between healthful and less healthful consumption patterns and cognitive measures [23,54,55,56]. The exact dietary mechanisms that influence brain function remain unclear, but research demonstrates a link between foods rich in refined carbohydrates, such as SSBs and sweet snacks, with cognitive dysfunction [57]. Evidence from animal and human studies suggests that dietary factors can affect brain processes by influencing neurotransmitter pathway regulation, synaptic transmission, membrane fluidity, and signal transduction pathways [14]. While lack of healthful diets in children’s daily lives is shown to interfere with learning processes or manifest in behaviors that hinder academic performance [26], the impact of diet on cognition may be more pronounced early in life or when studied longitudinally over extended time periods [10]. 

Surprisingly, we found no association between healthful food consumption and improved academic achievement. It is possible that consumption of unhealthy food items is more tightly linked to academic outcomes than that of healthier food items, or that the positive effects of healthful food intake are outweighed by the negative effects of less healthful foods. The FLEX FFQ, though shown to be a valid measure of child eating patterns within this diverse group of children, may not fully capture food groups positively associated with academic performance. It is also possible that the impact of healthful foods such as fruits and vegetables on brain function is most apparent after long-term consumption. This is supported by a systematic review of longitudinal studies demonstrating small, positive relationships between healthful diets during infancy and IQ later in childhood [23]. Paradoxically, we found an inverse association with fruit intake and English standardized test scores. It is possible that fruit intake may be a marker for overall sweet consumption, an idea that is supported by positive correlations between fruit intake and sweet snack/SSB intake in our sample (data not shown).

This study is limited by the use of an FFQ, which did not allow total calories to be controlled. The FFQ was designed to assess a subset of healthful foods (fruits, vegetables, unsweetened beverages) and less healthful foods (sweet and salty snack foods, SSBs) that are under- and overconsumed, respectively, within adolescent populations in the US [37]. Furthermore, since children often eat limited types and amounts of food and may have irregular eating patterns, using servings-per-day as a unit of measurement may introduce bias. There were a large number of observations of reported dietary intake that were implausible and were dropped from the analysis. However, some level of over- and under-reporting was expected, since the FFQ was child-administered and did not measure total diets. In addition, the FLEX FFQ was found to be a valid measure of intake within the FLEX study population. Although diverse, the sample was limited to children attending schools in lower-income Massachusetts public school districts and may not be generalizable to broader populations. English language learners may perform differently on cognitive tests, particularly Stroop, which measures one’s inhibitory control (for example, if one sees the word “blue” written in green, it may be easier for an English language learner to read the word “blue”). Results regarding the relationship between dietary intake variables and academic achievement were limited to the subset of 4th graders who were eligible to take the MCAS exam. Finally, although we were limited by the cross-sectional design of this study, it allowed for observation of short-term dietary associations. Establishing the link between dietary patterns over time and cognitive health is an important next step. 

The strengths of this study include a large and diverse sample of children from racial/ethnic minorities and low-socioeconomic status backgrounds, with academic achievement measures encompassing both cognitive tests and standardized test scores. The FLEX study utilized validated measures of diet and cognition, collected BMI values, and objectively measured physical activity levels; previous studies have often relied on self-report for these variables [23,29]. We were able to control for additional confounding factors, including English language learner status in diet and academic achievement models and parental education in all models. This study examined the possible link between intake of food groups and patterns of healthful and less healthful foods that encompass the range of nutrients, or lack thereof, and performance and cognition. 

## 5. Conclusions

This analysis provides further evidence regarding the association between diet, cognition, and academic achievement in diverse populations. Greater consumption of less healthful food groups was associated with significantly lower standardized math and English language arts test scores, suggesting that further recommendations should focus not only on encouraging healthful food group intake but also on minimizing foods that are less healthful to have a greater impact on academic success. Future work is necessary to corroborate these results. Our study did not demonstrate an association between dietary intake and cognitive measures, highlighting the need for further research to inform policies surrounding school food programs and nutrition messaging targeted toward youth and their families. Future research should focus on dietary patterns that are interpretable by general audiences, including children, parents, school staff, and policy-makers, to offer easily translatable nutrition advice.

## Figures and Tables

**Table 1:** Baseline demographic characteristics along with cognitive and standardized test scores (MCAS) ^a,b^ of the Fueling Learning Through Exercise study population.

	Entire Sample (*n* = 868)		MCAS Only (*n* = 430)	
Sex, *n* (%)				
Boys	376	(43.3)	198	(46.0)
Girls	492	(56.7)	232	(54.0)
Age (years), mean (sd)	8.7	(0.7)	9.16	(0.4)
Grade, *n* (%)				
3rd grade	397	(45.7)	–	–
4th grade	471	(54.3)	430	(100)
BMI for age Z-score, *n* (%) ^c^				
Underweight	10	(1.2)	5	(1.2)
Healthy weight	506	(58.3)	244	(56.7)
Overweight	166	(19.1)	95	(22.1)
Obese	186	(21.4)	86	(20.0)
Race/ethnicity, *n* (%)				
Non-Hispanic white	288	(33.2)	158	(36.7)
Non-Hispanic black	72	(8.3)	30	(7.0)
Hispanic	227	(26.2)	113	(26.3)
Multiracial/Asian/American Indian/other	148	(17.1)	59	(13.7)
Declined to respond	133	(15.3)	70	(16.3)
English language learner, *n* (%) ^d^	–	–	92	(21.4)
Maternal education, *n* (%)				
High school degree or less	292	(33.6)	133	(30.9)
Some college or associate degree	238	(27.4)	128	(29.8)
Bachelor’s degree or above	261	(30.1)	129	(30.0)
Declined to respond	77	(8.9)	40	(9.3)
Paternal education, *n* (%)				
High school degree or less	374	(43.1)	168	(39.1)
Some college or associate degree	180	(20.7)	95	(22.1)
Bachelor’s degree or above	206	(23.7)	108	(25.1)
Declined to respond	108	(12.4)	59	(13.7)
Cognitive and academic test scores				
Digit Span forwards, mean (sd)	4.7	(1.1)	4.8	(1.1)
Digit Span backwards, mean (sd)	2.8	(1.0)	2.9	(1.0)
Stroop ratio, mean (sd) ^e^	0.4	(0.1)	0.4	(0.1)
MCAS mathematics score, *n* (%)				
Warning	–	–	45	(10.5)
Needs improvement	–	–	80	(18.6)
Proficient	–	–	164	(38.1)
Advanced	–	–	141	(32.8)
MCAS English score, *n* (%)				
Warning	–	–	24	(5.6)
Needs improvement	–	–	156	(36.3)
Proficient	–	–	197	(45.8)
Advanced	–	–	53	(12.3)

^a^ Massachusetts Comprehensive Assessment System. ^b^ MCAS assessments only available for 4th- grade students at baseline. ^c^ Defined according to Centers for Disease Control and Prevention cut points as underweight, <5th percentile; normal weight, 5th–85th percentile; overweight, 85th–95th percentile; obese, ≥95th percentile. ^d^ Information regarding English language learner status was collected during MCAS testing and was only available for those 4th-grade students who were administered the exam. ^e^ Ratio of incongruent to congruent score. BMI: body mass index.

**Table 2:** Mean servings of foods consumed daily, as reported by the Fueling Learning Through Exercise (FLEX) Food Frequency Questionnaire (*n* = 868).

Healthful Foods	Mean	SD
Fruits	1.9	1.7
Vegetables	1.4	1.3
Non-sugar-sweetened beverages (total)	2.6	1.1
Water	1.2	0.5
Milk	1.0	0.8
Fruit juice	0.4	0.4
Less healthful foods		
Sweet snacks	1.6	1.7
Salty snacks	1.4	1.4
Sweetened beverages (total)	0.8	0.8
Soft drinks	0.3	0.5
Energy drinks	0.2	0.3
Flavored juice drinks	0.3	0.4

**Table 3:** Multiple Poisson regression models predicting the Digit Span scores by intake of food and beverage and combined food group servings in the FLEX study population (*n* = 868).

	Digit Span Forwards ^a^			Digit Span Backwards ^a^		
Dietary Intake	RR	*p*-Value	95% CI	RR	*p*-Value	95% CI
Healthful foods ^b^	1.001	0.821	0.991, 1.011	0.997	0.707	0.984, 1.011
Less healthful foods ^c^	0.993	0.159	0.982, 1.003	0.988	0.087	0.975, 1.002
Individual food group intake						
Fruits	1.001	0.884	0.978, 1.026	0.985	0.348	0.953, 1.017
Vegetables	1.001	0.928	0.973, 1.031	1.018	0.353	0.980, 1.057
Salty snacks	0.998	0.912	0.968, 1.030	0.984	0.043	0.944, 1.025
Sweet snacks	0.988	0.354	0.964, 1.013	0.997	0.873	0.965, 1.031
Non-sugar-sweetened beverages ^d^	0.999	0.948	0.969, 1.030	0.993	0.718	0.954, 1.033
Sugar-sweetened beverages ^e^	0.994	0.802	0.949, 1.040	0.979	0.488	0.921, 1.040

^a^ Adjusted for age in months. ^b^ Including fruits, vegetables, and non-sugar-sweetened beverages. ^c^ Including sweet snacks, salty snacks, and sugar-sweetened beverages. ^d^ Including water, milk, and 100% fruit juice. ^e^ Including sodas, energy drinks, and juice drinks.

**Table 4:** Multiple linear regression predicting Stroop ratio ^a^ by intake of food and beverage group servings in the FLEX study population (*n* = 806).

	Adjusted Model ^b^		
Dietary Intake	β	*p*-Value	95% CI
Healthful foods ^c^	0.000	0.665	−0.004, 0.002
Less healthful foods ^d^	0.000	0.941	−0.003, 0.003
Individual food group intake			
Fruits	−0.007	0.084	−0.014, 0.001
Vegetables	0.005	0.234	−0.003, 0.014
Salty snacks	0.009	0.060	−0.001, 0.018
Sweet snacks	−0.002	0.526	−0.001, 0.005
Non-sugar-sweetened beverages ^e^	0.003	0.221	−0.006, 0.012
Sugar-sweetened beverages ^f^	−0.008	0.221	−0.022, 0.004

^a^ Ratio of congruent to incongruent Stroop test score. ^b^ Adjusted for BMI Z-score and moderate-to-vigorous daily physical activity levels. ^c^ Including fruits, vegetables, and non-sugar-sweetened beverages. ^d^ Including sweet snacks, salty snacks, and sugar-sweetened beverages. ^e^ Including water, milk, and 100% fruit juice. ^f^ Including sodas, energy drinks, and juice drinks.

**Table 5:** Ordered logistic regression models predicting Massachusetts Comprehensive Assessment System mathematics and English language arts score by individual food group intake in the FLEX study population (*n* = 430).

	Mathematics Score ^a^			English Language Arts Score ^b^		
Dietary Intake	OR	*p*-Value	95% CI	OR	*p*-Value	95% CI
Healthful foods ^c^	0.976	0.531	0.905, 1.053	0.959	0.295	0.886, 1.038
Less healthful foods ^d^	0.905	0.014	0.836, 0.980	0.868	0.001	0.798, 0.943
Individual food group intake						
Fruits	0.935	0.457	0.782, 1.117	0.748	0.003	0.618, 0.906
Vegetables	0.941	0.511	0.783, 1.130	1.104	0.333	0.904, 1.347
Salty snacks	1.001	0.990	0.794, 1.263	1.142	0.292	0.892, 1.460
Sweet snacks	0.848	0.082	0.704. 1.021	0.720	0.001	0.591, 0.876
Non-sugar-sweetened beverages ^e^	1.105	0.354	0.894, 1.366	1.152	0.204	0.926, 1.433
Sugar-sweetened beverages ^f^	0.865	0.408	0.614, 1.219	0.978	0.902	0.690, 1.387

^a^ Adjusted for sex, English language learner status, enrollment in individualized education plan, father’s education level, and BMI-z score. ^b^ Adjusted for English language learner status, enrollment in individualized education plan and mother’s education level. ^c^ Including fruits, vegetables, and non-sugar-sweetened beverages. ^d^ Including sweet snacks, salty snacks, and sugar-sweetened beverages. ^e^ Including water, milk, and 100% fruit juice. ^f^ Including sodas, energy drinks, and juice drinks.

## Data Availability

The datasets used and/or analyzed during the current study are available from the corresponding author on reasonable request.
